# Heritability of dispersal‐related larval traits in the clown anemonefish *Amphiprion percula*

**DOI:** 10.1002/ece3.9541

**Published:** 2022-11-27

**Authors:** E Schlatter, CaitLynn Klawon, Colleen Webb, Peter Buston

**Affiliations:** ^1^ Boston University Boston Massachusetts USA; ^2^ Colorado State University Fort Collins Colorado USA; ^3^ Present address: University of California at Davis Davis California USA

**Keywords:** dispersal evolution, heritability, intraspecific variation, marine larval dispersal

## Abstract

A major goal of marine ecology is to identify the drivers of variation in larval dispersal. Larval traits are emerging as an important potential source of variation in dispersal outcomes, but little is known about how the evolution of these traits might shape dispersal patterns. Here, we consider the potential for adaptive evolution in two possibly dispersal‐related traits by quantifying the heritability of larval size and swimming speed in the clown anemonefish (*Amphiprion percula*). Using a laboratory population of wild‐caught *A. percula*, we measured the size and swimming speed of larvae from 24 half‐sibling families. Phenotypic variance was partitioned into genetic and environmental components using a linear mixed‐effects model. Importantly, by including half‐siblings in the breeding design, we ensured that our estimates of genetic variance do not include nonheritable effects shared by clutches of full‐siblings, which could lead to significant overestimates of heritability. We find unequivocal evidence for the heritability of larval body size (estimated between 0.21 and 0.34) and equivocal evidence for the heritability of swimming speed (between 0.05 and 0.19 depending on the choice of prior). From a methodological perspective, this work demonstrates the importance of evaluating sensitivity to prior distribution in Bayesian analysis. From a biological perspective, it advances our understanding of potential dispersal‐related larval traits by quantifying the extent to which they can be inherited and thus have the potential for adaptive evolution.

## Graphical abstract

A major goal of marine ecology is to identify the drivers of variation in larval dispersal. Here, we investigate the heritability of two key larval dispersal traits, body size and swimming speed, in *Amphiprion percula*, a model species for studies of coral reef fish dispersal. We find unequivocal evidence for heritability of larval body size (estimated between 0.21 and 0.34), and equivocal evidence for heritability of swimming speed (between 0.05 and 0.19 depending on the choice of prior).

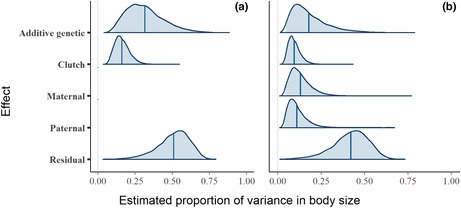

## 1 INTRODUCTION

A major goal of marine ecology is to identify drivers of variation in patterns of larval dispersal. Most marine fishes and invertebrates undergo early development in open water, sometimes moving long distances from their origin in the process. For many species, this larval dispersal phase is the primary driver of population connectivity and an essential component of spatial structure (Cowen et al., 2007; Dedrick et al., 2021). Understanding the causes and consequences of larval dispersal not only advances basic knowledge of marine ecology, but it also informs the design of effective, connected networks of marine reserves—widely‐used conservation tools that provide refuge to fished species and improve ecosystem resilience (Green et al., 2015; Hamilton et al., 2021; Krueck et al., 2017).

As data accumulate on patterns of marine larval dispersal, the extent of intra‐ and interspecific variation is becoming clear. Among species, the scale of dispersal varies widely: Some species have average dispersal distances on the order of 10 km (Almany et al., 2017; Catalano et al., 2021), while others travel hundreds of kilometers on average (Simpson et al., 2014; Williamson et al., 2016). Dispersal ability may influence range size, speciation, and extinction rates within lineages (Alzate et al., 2019; Jablonski, 1986). Within a species, some individuals may disperse only meters while others travel tens of kilometers (D'Aloia et al., 2013, 2022), with different implications for demographics and evolution: Common, short‐distance dispersal plays an important role in local population dynamics, while rare, long‐distance events may help to maintain genetic connectivity and define the spatial scale at which population divergence occurs.

Although the drivers of this variation are not yet well‐understood, one key theme is the role of larval traits. Dispersing larvae were once thought to be passive, their movement determined entirely by the physical oceanographic properties of the environment (Roberts, 1997), but larvae have well‐developed swimming and navigational abilities with the potential to affect their dispersal trajectories from the time they hatch (Leis et al., 2011; Majoris et al., 2021; Paris et al., 2013). Two traits that have been suggested to have a role in dispersal outcomes are size and swimming speed. Among species, both traits are related to range size and population genetic structure (Bradbury et al., 2008; Nanninga & Manica, 2018) and the spatial scale of long‐distance dispersal (Majoris et al., 2019). Their relevance at the interspecific level indicates they might also explain intraspecific variation in dispersal. Less is known about the relationship between traits and dispersal within species, because of the difficulty of measuring traits and realized dispersal distances for individual larvae. However, some evidence is available. For example, individual variation in body size predicts performance and/or mortality at various stages of larval development (Nanninga & Berumen, 2014), and an empirically‐validated biophysical model with realistic assumptions about larval traits (including size and swimming ability) found a positive relationship between swimming speed and dispersal distance in grouper larvae (Burgess et al., 2021).

Although progress is being made on ecological questions about how a larva's traits may influence its dispersal, much less is known about how the evolution of these traits could shape, or be shaped by, dispersal patterns. An important starting point in understanding adaptive evolutionary potential is to quantify phenotypic variation in the trait of interest and identify the sources of that variation. In classical quantitative genetics, the response of a trait to selection is determined by its heritability (*h* ^*2*^): the proportion of phenotypic variance that is due to additive genetic effects (Fisher, 1918; Wright, 1921). In reality, the picture is much more complicated: the genetic background and environmental context in which a trait is found may have profound effects on its expression and the resulting phenotype and fitness of the organism (West‐Eberhard, 2003). At present, however, even basic information about the sources of phenotypic variance in larval dispersal traits is scarce. The few studies that quantify the heritability of size and/or swimming performance in marine fish larvae have found heritability values ranging from 0.10 to 0.65 (Bang et al., 2006; Gao & Munch, 2013; Garenc et al., 1998; Johnson et al., 2010). But a heritability estimate means little without additional context about how it was derived. Especially in highly fecund species like fishes (where breeding designs typically include clutches of many individuals that share the same environment and genetic inheritance), efforts to partition phenotypic variance can be confounded by parental, shared environmental, and genetic effects (Garenc et al., 1998), leading to potential overestimates of heritability. Through careful breeding design, some studies have been able to explicitly separate maternal, paternal, and/or common environmental effects (Bang et al., 2006; Gao & Munch, 2013; Johnson et al., 2010). But there is a need for additional studies of this type, particularly in species for which other information about dispersal is available.

Here, we begin to quantify the adaptive potential of dispersal‐related larval phenotypes in the clown anemonefish (*Amphiprion percula*), a reef fish that has been the focus of an enormous amount of work on marine larval dispersal (Almany et al., 2007, 2017; Buston et al., 2012; Majoris et al., 2019; Planes et al., 2009). The life‐history and reproductive behavior of this species indicate factors that we might expect to be particularly important sources of larval phenotypic variation. They live in small, stable groups that permanently inhabit an anemone (Elliott & Mariscal, 2001). Each group contains just one breeding pair, and the female lays demersal eggs that are fertilized by the male (Buston, 2004). Both parents tend to the eggs by oxygenating them and removing debris, although the male performs the majority of this activity (Barbasch & Buston, 2018). Parental care during the incubation period is crucial to proper development and hatching success (Barbasch et al., 2020). On hatching, larvae exhibit extensive phenotypic variation in larval size and swimming speed (Majoris et al., 2019) and dispersal distances (Almany et al., 2017). When considering phenotypic variance in larvae, therefore, we must separate the influences of environment (e.g., habitat quality in the anemone where the embryo is located), parents (e.g., maternal provisioning and parental care of eggs), and genetics.

Here, we take a quantitative genetic approach to disentangling genetic, parental, and environmental sources of phenotypic variance in dispersal‐related larval traits. We use an experimental breeding design consisting of full‐ and half‐siblings and a statistical model known as the “animal model” (Kruuk, 2004; Lynch & Walsh, 1998): a linear mixed‐modeling approach that uses data about the relatedness and phenotypes of individuals to estimate the magnitude of the additive genetic component of phenotypic variance. The results provide estimates of the heritability of larval size and swimming speed in our study population of *A. percula*.

## 2 METHODS

### 2.1 Study population

The larvae in this study were offspring of parents from an established laboratory population of *A. percula*. Parents were caught from natural populations in Papua New Guinea and supplied by Quality Marine in 2010–2011. These wild‐caught fish were collected as nonbreeders: considered to be a sustainable fishing practice, as nonbreeders do not contribute to population growth and are readily replaced by newly‐settling larvae (Buston, 2003, 2004; Planes et al., 2009). Individuals were randomly placed in pairs upon arrival in the lab, where they established dominance and began breeding.

The laboratory population of breeding pairs was maintained at Boston University in accordance with Institutional Animal Care and Use protocol #17–001. Each breeding pair was housed in its own 120‐L tank, and pumps supplied a continuous flow of recirculating salt water at a rate of approximately 16,600 L h^−1^. Water quality was kept as constant as possible and similar to conditions in the fish's native reef habitat: pH = 8.0–8.3, temperature = 27–28°C, salinity = 33–35 ppt, with the lighting supplied by two T5 24 W bulbs with spectra that mimic the natural reef environment. Tanks contained sand on the bottom, a 15 × 15 cm ceramic tile, an *Entacmaea quadricolor* anemone, and an approximately 10 × 10 cm rock for habitat. Fish were fed commercial pellets (New Life Spectrum; New Life International, Inc.) at approximately 24 pellets per pair per day, a food ration that results in pairs reproducing at a rate and quantity consistent with the reproduction observed in the field (Barbasch et al., 2020).

### 2.2 Breeding design

**FIGURE 1:**
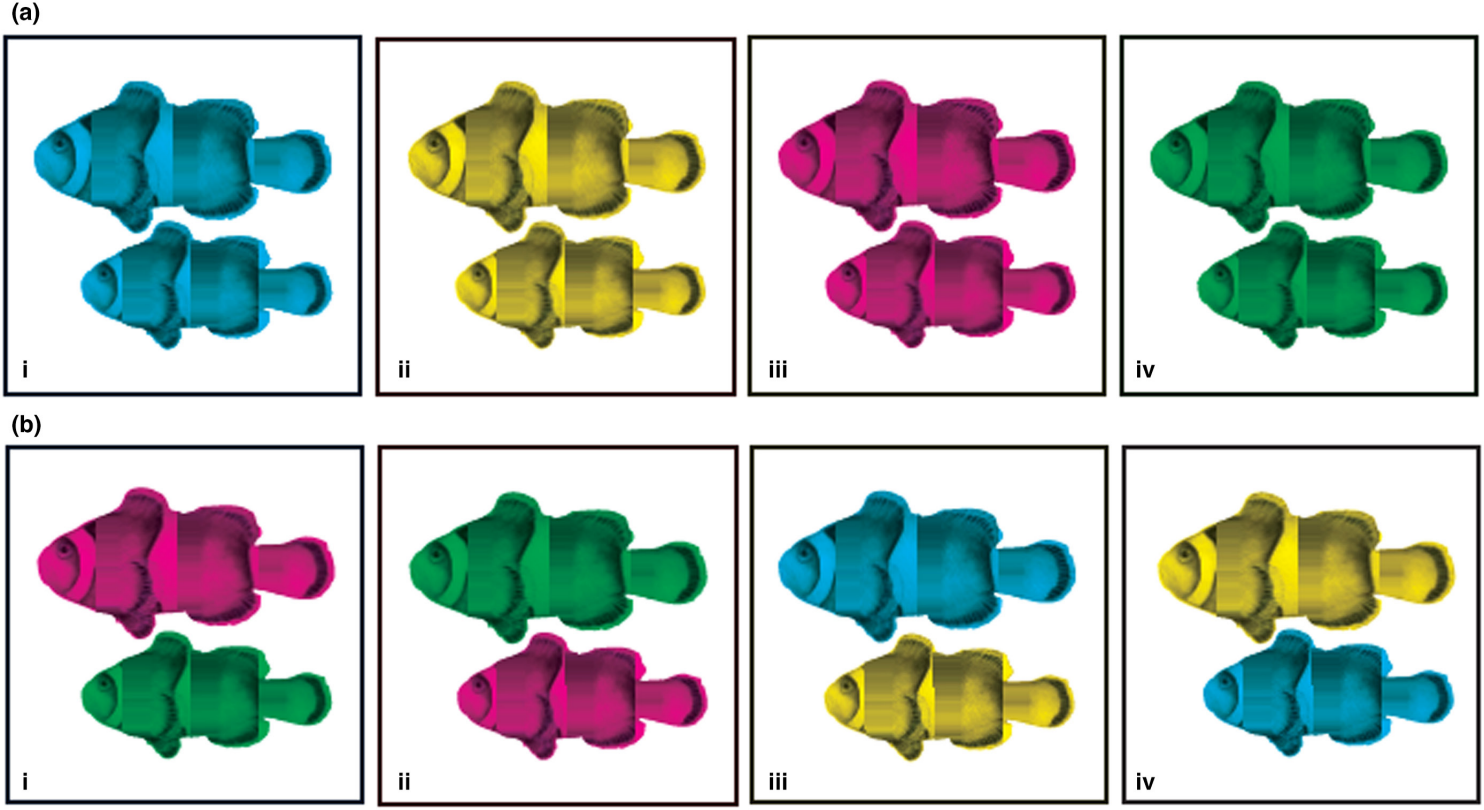
Larval trait heritability breeding design. The first round of parental pairings is shown in row (a), and the second (following mate and tank switches) is in row (b). Each tank is identified by a Roman numeral i–iv, and individual fish are identified by size and color. Clownfish drawn by Rebecca Branconi.

To estimate the additive genetic variance in larval traits, we collected trait data from full‐ and half‐siblings from 12 mothers and 12 fathers. The 24 parents were established in breeding pairs prior to the beginning of the study. We first sampled 30 offspring from each pair (Figure 1a) and measured their size and swimming speed as described below. Typically this could be accomplished with one clutch, but if fewer than 30 larvae from a clutch were available, additional larvae from a later clutch by the same parents were measured, for a total of 30 larvae per breeding pair (360 larvae total).

Next, the members of each breeding pair were recombined and moved among tanks so that each fish was paired with a new partner and located in a new tank (Figure 1b). To minimize potential aggression, each female was paired with a new male of the same size as her previous partner (±1 mm) (Wong et al., 2016). Newly formed pairs were allowed to breed undisturbed twice because the first clutches of new pairs often fail (Buston & Elith, 2011), and 30 larvae from their third clutch or later were sampled as above. The half‐siblings generated by this design are crucial: Without them, our estimates of genetic variance would include nonheritable effects shared by full‐siblings, and could greatly overestimate the heritability of larval traits and thus their adaptive potential.

### 2.3 Acquisition of larvae

Reproduction of breeding pairs was monitored daily so that the average incubation period of each pair and the age of all eggs laid were known, allowing us to reliably predict when any clutch would hatch. The incubation period varied from 7 to 10 days in the lab, and this was recorded for each clutch and used in the statistical model (see below) to account for potential developmental differences among larvae due to their length of incubation. Clutches of eggs were collected from breeding pairs the night before hatching and moved to separate 20‐gallon tanks maintained at 34 ppt salinity and 27–28°C. Hatching tank water was tinted with *Nannochloropsis* algae and provisioned with *Brachionus rotundiformis* rotifers at a concentration of 10 rotifers ml^−1^. Eggs were agitated with a gentle flow of air from an air stone diffuser to mimic parental care, and larvae that hatched overnight were sampled the next morning.

We recorded clutch ID (*n*_*c*_ = 30), maternal ID (*n*_*d*_ = 12), and paternal ID (*n*_*s*_ = 12) for each larva, and these were included as random effects in the statistical model (see below). Clutch ID was included to account for similarities among clutch‐mates due to shared conditions experienced during development and early life. Maternal ID represents any effect of the mother beyond the additive effect of the alleles passed down to her offspring; this includes factors such as nutritional state (and the consequent amount and quality of egg provisioning) and inherited epigenetic patterns of gene expression. Similarly, paternal ID represents any effect of the father beyond the additive genetic; we expect this to be particularly important in clownfish because males provide extensive parental care to their eggs (Barbasch & Buston, 2018), which influences the eggs' survival (Barbasch et al., 2020).

### 2.4 Larval trait measurements

To measure critical swimming speed, we used a single‐channel swimming flume, described in detail elsewhere (Majoris et al., 2019). Larvae were removed from their hatching tank in a 90‐ml sample cup immediately before each trial and placed into the flume chamber using a large‐bore pipette. Once a larva was introduced into the flume, a 2‐min acclimation period began at a water velocity of <1 cm s^−1^. Larvae that exited the chamber or exhibited irregular behavior during the acclimation period (58 larvae overall) were removed from the experiment, and additional larvae were trialed to reach a total of 30 successful trials per parental pair. After acclimation, the water velocity was increased to 2 cm s^−1^ and maintained for 2 min, then increased by an additional 2 cm s^−1^ every 2 min thereafter. The trial ended when the larva was unable to successfully swim against the current and was expelled out the back of the flume. Critical swimming speed was calculated as

$$U_{\text{crit}}=U+\left(t*\frac{2\;\mathrm{cm}\;s^{-1}}{2\;\min}\right)$$

where *U* is the penultimate speed (in cm s^−1^), and *t* equals the time (in minutes) the larva swam at the final speed (Brett, 1964).

To measure larval size, each larva was removed from the flume after the swim trial and photographed with a Canon 60D digital SLR camera mounted on a Zeiss Stemi 2000‐C stereo dissection microscope. Standard length (SL)—defined for preflexion larvae as the distance from the tip of the snout to the end of the notochord (Roux et al., 2019)—was measured from photographs using ImageJ (NIH). Standard length is a standard measure of the larval size and is closely correlated with other measurements of body size (Parichy et al., 2009).

### 2.5 Statistical analyses

To partition the observed phenotypic variance in larval size and swimming speed into genetic and environmental components, we used a linear mixed‐modeling technique for estimating quantitative genetic parameters, known as the “animal model” (Kruuk, 2004). This method was chosen over others (such as the classic full‐sib, half‐sib, or North Carolina breeding design structures and their accompanying variance estimates) because it allows us to estimate the magnitude of additive genetic variance in each trait using all available data on the relatedness of individuals, it can include fixed and random effects, and it provides estimates of the contribution of each random component to the total variance.

A bivariate model considering the larval size and swimming speed simultaneously indicated no genetic covariance between the two traits (see Appendix S1), but MCMC algorithm convergence was not satisfactory for all variance components. Therefore, to facilitate convergence and simplify the interpretation of results, we proceeded to analyze the two traits separately. For each trait, the model structure was given by:

$$y=\mu +X\beta +a+Z_{c}c+Z_{d}d+Z_{s}s+r$$

where **y** is a vector of larval trait values with *n* = 688 entries (32 larvae were missing either size or swimming speed data and were excluded from the analysis), and μ is the mean trait value over all larvae. **X** is the *n*‐vector of incubation times and β is the effect of incubation time. The *n*‐vector **a** contains the additive genetic effects; **a** has a multivariate normal distribution with a variance–covariance structure **G** = $\sigma_{A}^{2}$ **A**, where $\sigma_{A}^{2}$ is the additive genetic variance and **A** is the *n × n* relatedness matrix among larvae (e.g., *A*_*ij*_ = 0.5 if larvae *i* and *j* are full‐siblings). Each **Z**_**i**_ is an incidence matrix relating larvae to the corresponding vector of random effects **c** (clutch), **d** (maternal ID), or **s** (paternal ID). The residual term **r** captures all contributions to phenotype not explicitly accounted for by other factors: This includes environmental sources of variation among members of the same clutch, as well as nonadditive genetic effects (dominance, epistasis, and their interactions). Vectors **c**, **d**, **s**, and **r** are normally distributed with variances $\sigma_{C}^{2},\sigma_{D}^{2},\sigma_{S}^{2}$, and $\sigma_{R}^{2}$, respectively, and covariances equal to zero.

We also fit a second, reduced model that did not include parental effects (the model structure was given by **y** = μ + **X**β + **a** + **Z**_**c**_ **c** + **r**). The reason for considering both models is that, in our breeding design, maternal ID, paternal ID, and additive genetic effect are highly collinear. All larvae in our sample that share genes (i.e., there is a positive covariance between their additive genetic effects) also share maternal ID, paternal ID, or both. This means that the full model may inaccurately attribute additive genetic variance to maternal or paternal ID, while the reduced model almost certainly attributes some parental effects (if they exist) to the additive genetic effect. Examining both models gives us a more realistic range for the strength of the additive genetic effect in this population.

Model fitting was performed using the MCMCglmm package (Hadfield, 2010) in R (R Core Team, 2021). The Bayesian framework provides posterior estimates of the distribution of each parameter of interest; an additional advantage is that calculating functions of these parameters (such as heritability) is straightforward. Each model was run in three chains of 1,000,000 iterations with a burn‐in of 10,000 and thinning interval of 100. Convergence was assessed using the Gelman‐Rubin diagnostic (Brooks & Gelman, 1998), which was below 1.03 in all cases. Scripts and data for all analyses are available at github.com/eschlatter/Larval_trait_h2.

We chose default prior distributions to represent the hypothesis that the total phenotypic variance was distributed equally among variance components. For each of the three variance components (or five, in the parental effects model) we used a diffuse inverse gamma prior with nu = 0.001 (Hadfield, 2010). The second parameter (*V*) of each prior was chosen such that the mode of the distribution was equal to 1/*k* of the total phenotypic variance, where *k* is the number of variance components. To test the sensitivity of our results to the choice of prior, we also fit each model using a second set of priors, representing the alternative hypothesis that our proposed effects do not contribute to phenotypic variance. These priors were close to zero for all variance components: parameter‐expanded inverse gamma distributions with *V* = 1, nu = 1000, *α*_*μ*_ = 0, *α*_*V*_ = 1. To quantify the impact of the alternative priors, we calculated the percentage deviation of the mean estimates from the alternative priors, compared with the mean estimates from the default priors (Depaoli et al., 2020).

## 3 RESULTS

### 3.1 Components of phenotypic variance: Larval body size

**FIGURE 2:**
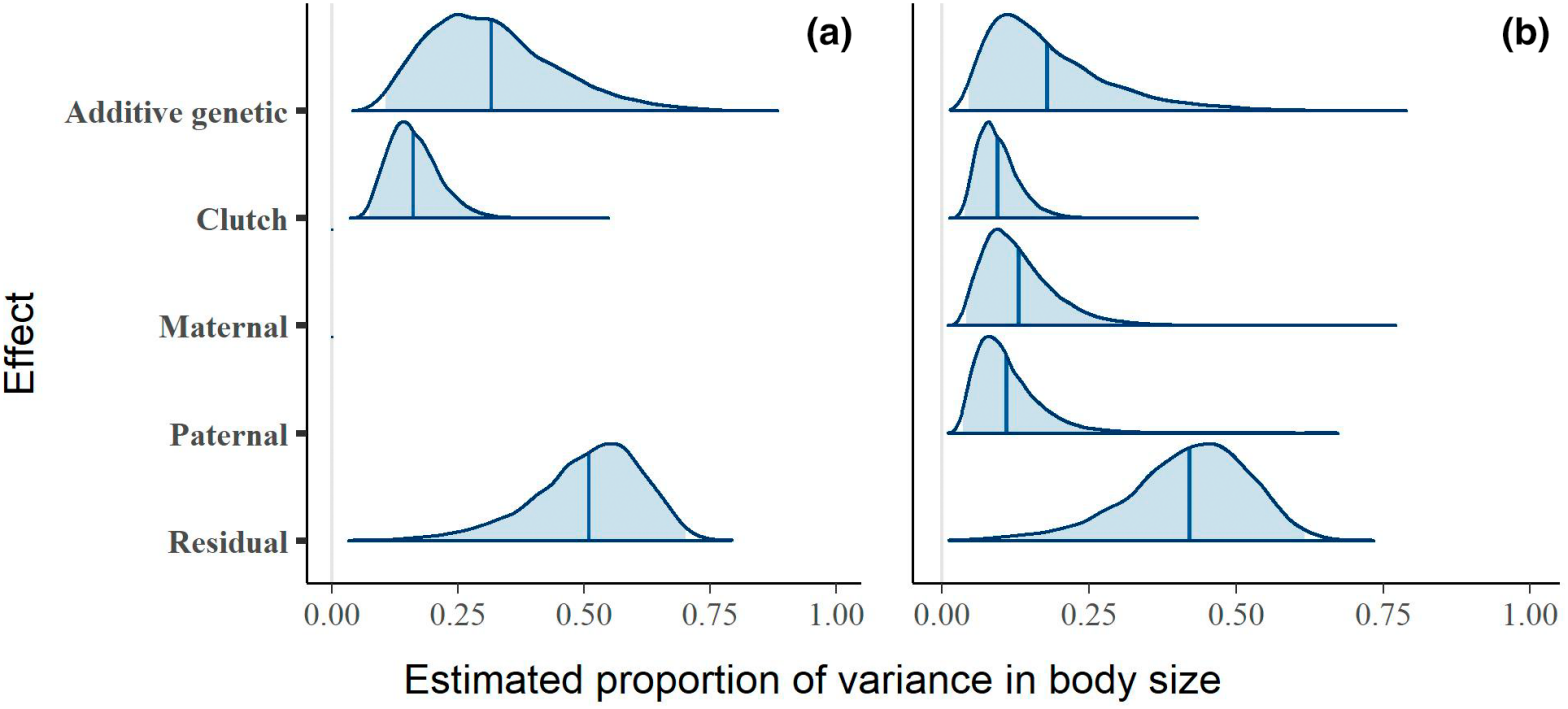
Posterior estimates of the relative magnitude of each component of phenotypic variance in larval body size, in models without (a) and with (b) parental effects. Shaded areas are 95% credible intervals, and dark lines are medians.

Larval standard length ranged from 3.92 to 4.61 mm, with a mean of 4.26 mm and a standard error of 0.005 mm. Total phenotypic variance in standard length was 0.016 mm^2^. The additive genetic variance estimated by our analysis was 0.0061 (0.0012–0.0130) (mean and 95% credible interval) in the model without parental effects and 0.0051 (0.0005–0.0130) in the model that included parental effects. Heritability of standard length was 34% (8–63%) in the model without parental effects (Figure 2a), or 21% (3–49%) when parental effects were included in the model (Figure 2b). The largest proportion of phenotypic variance in standard length was attributed to the residual: 49% (22–72%) for the model without parental effects included, and 41% (15–63%) with parental effects. The effect of the clutch was estimated at 17% (6–30%), or 10% (3–29%) with parental effects. Maternal effects accounted for 15% (3–31%) of total phenotypic variance, and paternal effects for 13% (2–28%).

Results from the models of body size were robust to the choice of prior, so only the estimates from the default priors are reported here. The estimate of heritability using the alternative set of priors deviated by −3% from the default estimate (or −4%, in the model with parental effects). For additional details on alternative priors, see the Appendix S1.

### 3.2 Components of phenotypic variance: Larval swimming speed

**FIGURE 3:**
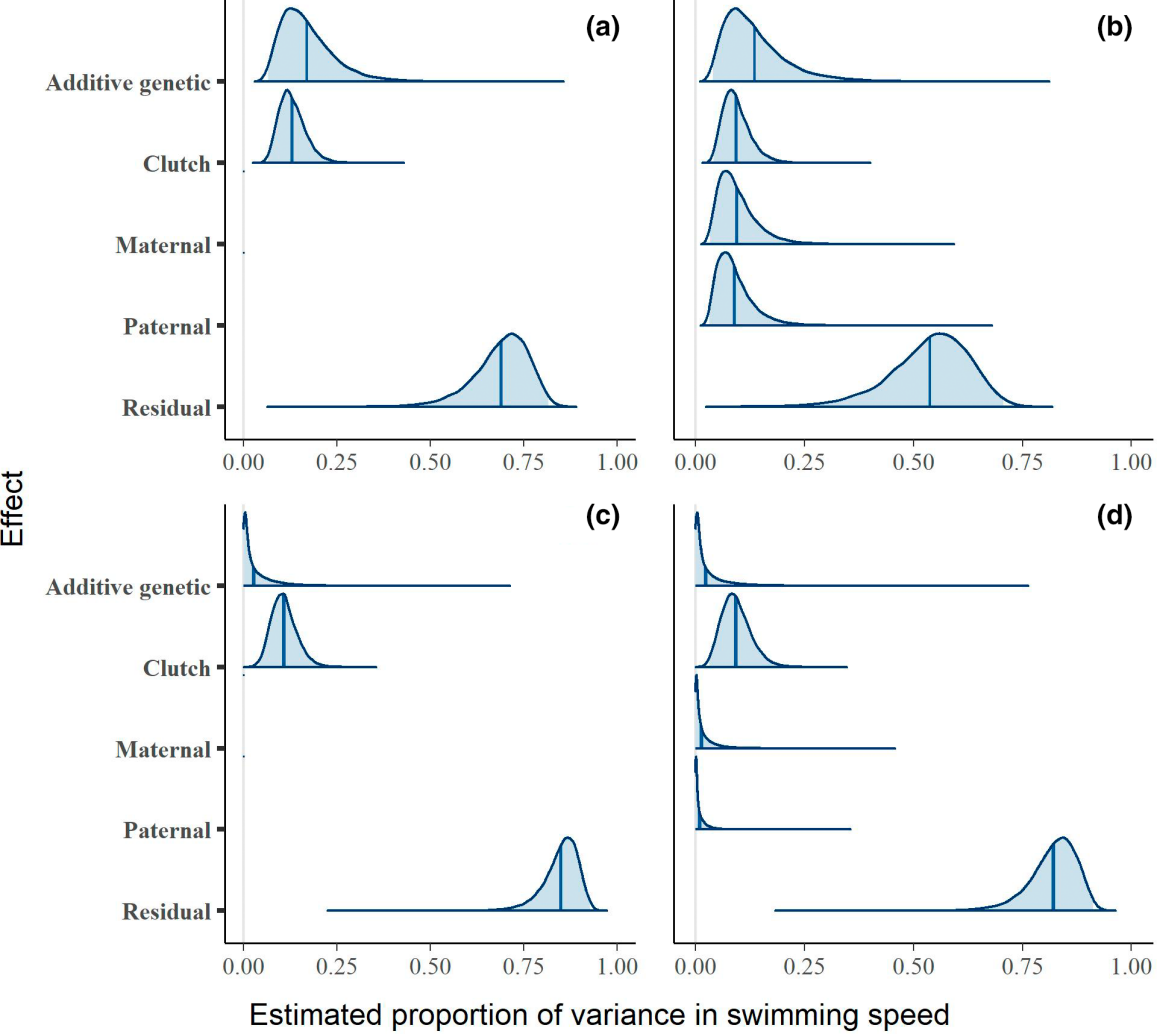
Posterior estimates of the relative magnitude of each component of phenotypic variance in larval swimming speed, in models without (a, c) and with (b, d) parental effects. Estimates in panels a and b were generated using the default priors, in which variance was distributed equally among all components. Panels c and d show estimates from the alternative priors, centered near zero for all components. Shaded areas are 95% credible intervals, and dark lines are medians.

Larval swimming speed measurements ranged from 0 to 9.67 cm s^−1^, with a mean of 3.91 cm s^−1^ and a standard error of 0.09 cm s^−1^. The total phenotypic variance in swimming speed was 4.67 cm^2^ s^−2^. The estimated additive genetic variance was 1.03 (0.23–2.24) cm^2^ s^−2^ (mean and 95% credible interval) in the model without parental effects, and 1.16 (0.14–2.98) when parental effects were included in the model. The heritability of swimming speed was 19% (47–83%) in the model without parental effects (Figure 3a), or 16% (29–73%) in the model with parental effects (Figure 3b). The largest amount of phenotypic variance in larval swimming speed was attributed to the residual: 67% (47–83%) or 52% (29–73%) with parental effects. Clutch accounted for 14% (6–23%) of variance or 10% (3–18%) with parental effects. Maternal effects contributed 11% (2–24%) to the total variance, and paternal effects contributed 10% (2–22%).

These results were sensitive to the choice of prior: The alternative set of priors, centered near zero for all variance components, resulted in a near‐zero estimate of heritability (Figure 3c,d): 5% (0–18%) without parental effects, or 5% (0–17%) with parental effects. This represents a −74% deviation from the default estimate (or −69%, in the model with parental effects). For further information on alternative priors, see the Appendix S1.

## 4 DISCUSSION

### 4.1 Sources of phenotypic variance in larval size and swimming speed

Our decomposition of phenotypic variance found evidence for genetic, parental, and environmental effects on the body size of newly‐hatched clownfish larvae. The heritability of larval body size was estimated to be 0.34 by the simpler model without parental effects, and 0.21 by the model that included parental effects. These estimates are within the range of those generated by other, similar studies of the heritability of larval body size in fishes (Bang et al., 2006; Gao & Munch, 2013; Johnson et al., 2010), and, more generally, of the heritability of dispersal‐related traits across species (Saastamoinen et al., 2018). Meanwhile, the presence of a clutch effect indicates the importance of the shared environment experienced by members of the same clutch during incubation and hatch. Within a clutch, a number of possible factors (discussed below) could be contributing to the residual variation among larvae.

The presence of maternal and paternal effects on larval body size is consistent with the few other studies that have sought to measure these effects in marine larval fishes. In Atlantic silversides, maternal effects on larval body size were greater than additive genetic effects (Gao & Munch, 2013); maternal effects on larval size at hatch have also been found in bicolor damselfish (Johnson et al., 2010). Significant effects of paternal identity on larval body size have been found in haddock (Rideout et al., 2004) and Atlantic herring (Bang et al., 2006). For clownfish, in particular, the presence of both maternal and paternal effects lines up with our expectations, given what we know about parental care in this species. Although females may have a unique opportunity for influence on the offspring's phenotype during egg production, male clownfish are responsible for the majority of the care of eggs once they are laid. Individual clownfish parents are consistent in the amount of care they provide to their eggs, and the amount of care varies depending on food availability (Barbasch & Buston, 2018). Parental care behaviors are crucial to the survival and hatching success of eggs, although their effect on larval traits such as body size is not yet known.

Because our breeding design included only a few of the many possible combinations of parents (each of the 12 females was mated with only two of the 12 males, and vice versa), there is high collinearity between maternal ID, paternal ID, and additive genetic effect, and thus considerable uncertainty in quantifying the effects of shared parents versus the effects of shared genes. The difference between estimates of heritability in the models with and without parental effects can be explained by the fact that similarities between siblings due to shared parents increase the overall resemblance among relatives in the dataset. In the simpler model, resemblance among relatives can be attributed to the additive genetic effect (for any full‐ or half‐siblings) or to the clutch effect (for members of the same clutch). In the more complex model, some of the resemblance among siblings that share a parent is accounted for by maternal or paternal effects, which decreases the amount of resemblance that is attributed to their shared genes. A breeding design that included more mates per parent, or relatives that do not share a parent (such as cousins), would help to more precisely quantify the relative contributions of parental and genetic effects.

Our evidence for genetic variance in larval swimming speed is equivocal. Although our default estimates (heritability of 0.19, or 0.15 from the model including parental effects) indicated a substantial role for additive genetic variance in larval swimming speed, those estimates were sensitive to the prior used to generate them. Our alternative prior yielded heritability estimates very close to zero. Since we do not have an a priori reason to be much more confident in the default prior than in the alternative, we can conclude only that, if the genetic variance in larval swimming speed exists in this population, our breeding design was unable to consistently detect it. This could perhaps be remedied by the modifications to the breeding design described above (e.g., including more mates per parent). In any case, the magnitude of genetic variance in swimming speed (if it exists) is small in comparison to the genetic variance in larval body size that we were able to detect with the same breeding design and individuals. There are at least two possible reasons for this. Critical swimming speed, as a behavioral trait, will have more phenotypic variability than a morphological trait such as standard length due to variation in the internal state of the larva. Genetic variation, if it exists, will then be a smaller proportion of the total and will be harder to detect. Alternatively, selection can reduce genetic diversity, particularly in traits closely associated with fitness (Fisher, 1930). If swimming speed strongly affects a larva's fitness, then it may have undergone past selection, reducing or eliminating genetic variance.

We did not find either genetic or phenotypic covariance between size and swimming speed, after controlling for the effects of the clutch. Genetic covariance might be expected as the result of a fitness tradeoff between growth and swimming performance (Billerbeck et al., 2001; Ghalambor, 2003), or selection on groups of traits as a dispersal syndrome: e.g., in Anolis lizards (Calsbeek & Irschick, 2007). However, our result generally agrees with other quantitative genetic studies that have sought to measure a genetic relationship between size and/or growth and swimming speed in young aquatic and marine organisms (Hilbish et al., 1999; Watkins & McPeek, 2006 but see Mengistu et al., 2021 for a counterexample in aquacultured Nile tilapia). The lack of phenotypic covariance measured in our study might seem to be at odds with the robust positive relationship between size and swimming speed that has been established for larval fishes (Leis, 2010). However, previous work demonstrating this relationship is based on samples that cover a significant portion of the larval developmental period (e.g., Majoris et al., 2019). Our sample of larvae at 0 days post hatch (dph) lacks the ontogenetic variation that causes the relationship between size and swimming speed in many other studies, and the amount of phenotypic variation we measured in both traits is comparatively quite small.

### 4.2 Evolutionary implications

We do not yet fully understand the relationship between the traits measured in this study, other larval traits throughout ontogeny, and dispersal outcomes. To our knowledge, there are no data available on the relationship between a larva's size at 0 dph and its size or swimming ability later in development. But for larvae of all ages, it is known that different measures of body size are strongly correlated (Parichy et al., 2009), and that body size is associated with survival—although the direction and details of this association vary by species and context (Gleiber et al., 2020; Pepin, 2016). For later‐stage larvae, multiple types of evidence (discussed in the introduction) suggest a connection between size and/or swimming ability and dispersal outcomes—although other metrics of swimming speed, besides critical swimming speed, may be more relevant to dispersal (Leis, 2020). So, while gaps remain in our understanding, it is reasonable to postulate that larvae face selection pressures that vary depending on their body size at 0 dph, with a subsequent impact on dispersal.

Given the existence of such selection pressures, our finding of genetic variance in 0‐dph larval body size is significant because it is an indication of adaptive potential. Genetic variance, such as we have found here, is a necessary condition for selection to lead to adaptive evolutionary change. Our finding provides empirical support for a fundamental assumption made by many models of dispersal evolution: namely, that dispersal is a trait that can evolve in response to selection via a somewhat predictable inheritance from parents to offspring. Demonstrating the heritability of outcome‐based measures of dispersal, such as distance or propensity, is a complicated undertaking in marine species. But we can begin to put the pieces together with our finding of heritability in larval body size, combined with empirical and modeling evidence that indicates larval traits such as body size affect dispersal outcomes.

It is important to note that any measurement of heritability is specific to the population and environmental conditions in which it is measured: Both the numerator (genetic variance) and the denominator (total phenotypic variance) of the heritability quantity depend on the particular set of individuals whose traits were measured. In this lab‐based experiment, we expect the genetic diversity of the study population to be relatively realistic: All larvae were offspring of parents wild‐caught from various locations in Papua New Guinea. However, the difference between environmental conditions in the wild and those in the lab means that applying our results uncritically to wild populations would be a mistake. The relatively standardized conditions in the lab probably reduce overall environmental variance, thus inflating heritability estimates over what they would be in the wild. In addition, systematic differences between experimental and natural habitats (for instance, in the missing chemical cues from the surrounding reef) likely affect trait variation in ways we cannot predict, with unknown consequences for heritability. Although this means our results must be interpreted with caution, the fact that we were able to detect genetic variance in larval body size indicates that this trait has one of the prerequisites for evolutionary change in response to selection.

More work is needed to understand the implications of parental effects on evolution. Without the reliable mechanism of genetic transmission, a continuity of parental effects is not guaranteed from one generation to the next. Nevertheless, there are an increasing number of documented instances of inter‐ and transgenerational parental effects (Bell & Hellmann, 2019), suggesting that this source of phenotypic variance has the potential to be inherited across one or multiple generations, and therefore could evolve in response to selection (Bonduriansky & Day, 2009). Disentangling parental effects that can be inherited from those that cannot would require a multigenerational study of parental identity and larval phenotype. This could be complemented by work to understand the phenotypic, environmental, and genetic mechanisms by which parental traits influence their offspring's phenotype.

For both larval size and swimming speed, a large portion of the phenotypic variance we observed remains unexplained by our models. This leaves the question: What causes differences among individuals? One component of this unexplained variation is differences among siblings. Mechanistically, it is plausible that parents might differentially allocate resources within a clutch to generate a range of phenotypes: for instance, through maternal provisioning or the spatial positions of eggs within the clutch (Green et al., 2006). This could be a selectively neutral process, or it could be adaptive as a form of bet‐hedging (Marshall et al., 2008) if it is advantageous for a parent to have some larvae disperse farther and others stay closer to home (Hamilton & May, 1977; Shaw et al., 2019).

### 4.3 Eco‐evolutionary dynamics of dispersal: *A. percula* as a model system

Dispersal plays a crucial role in both ecological and evolutionary processes. Particularly in the changing environment of today's oceans, where both demographics and selective forces are in flux, understanding the causes and consequences of dispersal requires an approach that can account for the significant overlap between ecological and evolutionary scales. With the foundations laid here and by others, *Amphiprion percula* represents a promising model system in which to investigate the eco‐evolutionary dynamics of larval dispersal traits. Extensive research has been conducted on *A. percula*'s larval dispersal patterns and their consequences for population connectivity (Almany et al., 2017; Pinsky et al., 2017). We know that data on the demographics of populations are crucial for correctly interpreting dispersal data (Pinsky et al., 2012), and this information is available for *A. percula* (Salles et al., 2016). The early development of its sister species, *Amphiprion ocellaris*, has been described in detail, providing information about the physical and sensory capabilities of dispersing larvae throughout ontogeny (Roux et al., 2019). Making quantitative predictions about eco‐evolutionary dynamics will also require detailed information about the genetic underpinnings of dispersal‐related traits, including their polygenic nature, genetic covariance among traits, and GxE interactions (Saastamoinen et al., 2018). Tackling these questions will require a high‐quality reference genome—which is already available for *A. percula* (Lehmann et al., 2018)—and other methods such as genome‐wide association studies and gene ontology studies to understand what genes are involved in dispersal‐related traits and how they interact with each other and with the environment. Our finding of significant heritability of larval body size in *A. percula* lays the foundation for these more detailed investigations.

## AUTHOR CONTRIBUTIONS

**E Schlatter:** Conceptualization (equal); formal analysis (lead); funding acquisition (equal); investigation (lead); writing – original draft (lead); writing – review and editing (equal). **CaitLynn Klawon:** Formal analysis (supporting); funding acquisition (equal); investigation (supporting); writing – review and editing (equal). **Colleen Webb:** Conceptualization (equal); formal analysis (supporting); funding acquisition (equal); supervision (equal); writing – review and editing (equal). **Peter Buston:** Conceptualization (equal); formal analysis (supporting); funding acquisition (equal); investigation (supporting); resources (lead); supervision (equal); writing – original draft (supporting); writing – review and editing (equal).

### OPEN RESEARCH BADGES

This article has earned the Open Data badge only. Data, materials and the preregistered design and analysis plan are available at https://doi.org/10.6084/m9.figshare.19623825.v1.

## Supporting information


Appendix S1

## Data Availability

Data are archived at https://doi.org/10.6084/m9.figshare.19623825.v1, and code for all analyses is available at github.com/eschlatter/Larval_trait_h2.

## References

[ece39541-bib-0001] Almany, G. R. , Berumen, L. M. , Thorrold, S. R. , Planes, S. , & Jones, G. P. (2007). Local replenishment of coral reef fish populations in a marine reserve. Science, 316, 742–744. 17478720 10.1126/science.1140597

[ece39541-bib-0002] Almany, G. R. , Planes, S. , Thorrold, S. R. , Berumen, M. L. , Bode, M. , Saenz‐Agudelo, P. , Bonin, M. C. , Frisch, A. J. , Harrison, H. B. , Messmer, V. , Nanninga, G. B. , Priest, M. A. , Srinivasan, M. , Sinclair‐Taylor, T. , Williamson, D. H. , & Jones, G. P. (2017). Larval fish dispersal in a coral‐reef seascape. Nature Ecology & Evolution, 1(6), 0148. 10.1038/s41559-017-0148 28812625

[ece39541-bib-0003] Alzate, A. , Janzen, T. , Bonte, D. , Rosindell, J. , & Etienne, R. S. (2019). A simple spatially explicit neutral model explains the range size distribution of reef fishes. Global Ecology and Biogeography, 28(7), 875–890. 10.1111/geb.12899

[ece39541-bib-0004] Bang, A. , Grønkjær, P. , Clemmesen, C. , & Høie, H. (2006). Parental effects on early life history traits of Atlantic herring (*Clupea harengus* L.) larvae. Journal of Experimental Marine Biology and Ecology, 334(1), 51–63. 10.1016/j.jembe.2006.01.003

[ece39541-bib-0005] Barbasch, T. A. , & Buston, P. M. (2018). Plasticity and personality of parental care in the clown anemonefish. Animal Behaviour, 136, 65–73. 10.1016/j.anbehav.2017.12.002

[ece39541-bib-0006] Barbasch, T. A. , Rueger, T. , Srinivasan, M. , Wong, M. Y. L. , Jonesnd, G. P. , & Buston, P. M. (2020). Substantial plasticity of reproduction and parental Care in Response to local resource availability. Oikos, 129, 1844–1855. 10.1111/oik.07674

[ece39541-bib-0007] Bell, A. , & Hellmann, J. (2019). An integrative framework for understanding the mechanisms and multigenerational consequences of transgenerational plasticity. Annual Review of Ecology, Evolution, and Systematics, 50, 97–118. 10.1146/annurev-ecolsys-110218-024613 PMC9427003 36046014

[ece39541-bib-0008] Billerbeck, J. M. , Lankford, T. E. , & Conover, D. O. (2001). Evolution of intrinsic growth and energy acquisition rates. I. Trade‐offs with swimming performance in *Menidia menidia* . Evolution, 55(9), 1863–1872. 10.1111/j.0014-3820.2001.tb00835.x 11681741

[ece39541-bib-0009] Bonduriansky, R. , & Day, T. (2009). Nongenetic inheritance and its evolutionary implications. Annual Review of Ecology, Evolution, and Systematics, 40(1), 103–125. 10.1146/annurev.ecolsys.39.110707.173441

[ece39541-bib-0010] Bradbury, I. R. , Laurel, B. , Snelgrove, P. V. R. , Bentzen, P. , & Campana, S. E. (2008). Global patterns in marine dispersal estimates: The influence of geography, taxonomic category and life history. Proceedings of the Royal Society B: Biological Sciences, 275(1644), 1803–1809. 10.1098/rspb.2008.0216 PMC2587791 18445556

[ece39541-bib-0011] Brett, J. R. (1964). The respiratory metabolism and swimming performance of young sockeye Salmon. Journal of the Fisheries Research Board of Canada, 21(5), 1183–1226. 10.1139/f64-103

[ece39541-bib-0012] Brooks, S. , & Gelman, A. (1998). General methods for monitoring convergence of iterative simulations. Journal of Computational and Graphical Statistics, 7(4), 434–455.

[ece39541-bib-0013] Burgess, S. C. , Bode, M. , Leis, J. M. , & Mason, L. B. (2021). Individual variation in marine larval‐fish swimming speed and the emergence of dispersal kernels. Oikos, 2022(3), 1–12. 10.1111/oik.08896

[ece39541-bib-0014] Buston, P. M. (2003). Forcible eviction and prevention of recruitment in the clown anemonefish. Behavioral Ecology, 14(4), 576–582. 10.1093/beheco/arg036

[ece39541-bib-0015] Buston, P. M. (2004). Does the presence of non‐breeders enhance the fitness of breeders? An experimental analysis in the clown anemonefish *Amphiprion percula* . Behavioral Ecology and Sociobiology, 57(1), 23–31. 10.1007/s00265-004-0833-2

[ece39541-bib-0016] Buston, P. M. , & Elith, J. (2011). Determinants of reproductive success in dominant pairs of clownfish: A boosted regression tree analysis. Journal of Animal Ecology, 80(3), 528–538. 10.1111/j.1365-2656.2011.01803.x 21284624

[ece39541-bib-0017] Buston, P. M. , Jones, G. P. , Planes, S. , & Thorrold, S. R. (2012). Probability of successful larval dispersal declines fivefold over 1 km in a coral reef fish. Proceedings of the Royal Society B: Biological Sciences, 279(1735), 1883–1888. 10.1098/rspb.2011.2041 PMC3311883 22158958

[ece39541-bib-0018] Calsbeek, R. , & Irschick, D. J. (2007). The quick and the dead: Correlational selection on morphology, performance, and habitat use in Island lizards. Evolution, 61(11), 2493–2503. 10.1111/j.1558-5646.2007.00206.x 17725626

[ece39541-bib-0019] Catalano, K. A. , Dedrick, A. G. , Stuart, M. R. , Puritz, J. B. , Montes, H. R. , & Pinsky, M. L. (2021). Quantifying dispersal variability among nearshore marine populations. Molecular Ecology, 30(10), 2366–2377. 10.1111/mec.15732 33197290

[ece39541-bib-0020] Cowen, R. , Gawarkiewicz, G. , Pineda, J. , Thorrold, S. , & Werner, F. (2007). Population connectivity in marine systems: An overview. Oceanography, 20(3), 14–21.

[ece39541-bib-0021] D'Aloia, C. C. , Bogdanowicz, S. , Andres, J. , & Buston, P. M. (2022). Population assignment tests uncover rare long‐distance marine larval dispersal events. Ecology, 103(1), e03559. 10.1002/ecy.3559 34653260

[ece39541-bib-0022] D'Aloia, C. C. , Bogdanowicz, S. M. , Majoris, J. E. , Harrison, R. G. , & Buston, P. M. (2013). Self‐recruitment in a Caribbean reef fish: A method for approximating dispersal kernels accounting for seascape. Molecular Ecology, 22(9), 2563–2572. 10.1111/mec.12274 23495725

[ece39541-bib-0023] Dedrick, A. G. , Catalano, K. A. , Stuart, M. R. , White, J. W. , Montes, H. R. , & Pinsky, M. L. (2021). Persistence of a reef fish metapopulation via network connectivity: Theory and data. Ecology Letters, 24(6), 1121–1132. 10.1111/ele.13721 33750002

[ece39541-bib-0024] Depaoli, S. , Winter, S. D. , & Visser, M. (2020). The importance of prior sensitivity analysis in Bayesian statistics: Demonstrations using an interactive shiny app. Frontiers in Psychology, 11, 608,045. 10.3389/fpsyg.2020.608045 33324306 PMC7721677

[ece39541-bib-0025] Elliott, J. K. , & Mariscal, R. N. (2001). Coexistence of nine anemonefish species: Differential host and habitat utilization, size and recruitment. Marine Biology, 138(1), 23–36. 10.1007/s002270000441

[ece39541-bib-0026] Fisher, R. A. (1918). The correlation between relatives on the supposition of mendelian inheritance. Transactions of the Royal Society of Edinburgh, 52(2), 399–433.

[ece39541-bib-0027] Fisher, R. A. (1930). The genetical theory of natural selection. Clarendon Press.

[ece39541-bib-0028] Gao, J. , & Munch, S. (2013). Genetic and maternal variation in early growth in the Atlantic silverside *Menidia menidia* . Marine Ecology Progress Series, 485, 211–222. 10.3354/meps10333

[ece39541-bib-0029] Garenc, C. , Silversides, F. G. , & Guderley, H. (1998). Burst swimming and its enzymatic correlates in the threespine stickleback (*Gasterosteus aculeatus*): Full‐sib heritabilities. Canadian Journal of Zoology, 76, 680–688.

[ece39541-bib-0030] Ghalambor, C. K. (2003). Multi‐trait selection, adaptation, and constraints on the evolution of burst swimming performance. Integrative and Comparative Biology, 43(3), 431–438. 10.1093/icb/43.3.431 21680451

[ece39541-bib-0031] Gleiber, M. , Sponaugle, S. , Robinson, K. , & Cowen, R. (2020). Food web constraints on larval growth in subtropical coral reef and pelagic fishes. Marine Ecology Progress Series, 650, 19–36. 10.3354/meps13217

[ece39541-bib-0032] Green, A. L. , Maypa, A. P. , Almany, G. R. , Rhodes, K. L. , Weeks, R. , Abesamis, R. A. , Gleason, M. G. , Mumby, P. J. , & White, A. T. (2015). Larval dispersal and movement patterns of coral reef fishes, and implications for marine reserve network design: Connectivity and marine reserves. Biological Reviews, 90(4), 1215–1247. 10.1111/brv.12155 25423947

[ece39541-bib-0033] Green, B. S. , Anthony, K. R. N. , & McCormick, M. I. (2006). Position of egg within a clutch is linked to size at hatching in a demersal tropical fish. Journal of Experimental Marine Biology and Ecology, 329(1), 144–152. 10.1016/j.jembe.2005.08.012

[ece39541-bib-0034] Hadfield, J. D. (2010). MCMC methods for multi‐response generalized linear mixed models: The MCMCglmm R package. Journal of Statistical Software, 33(2), 1–22. 10.18637/jss.v033.i02 20808728

[ece39541-bib-0035] Hamilton, R. J. , Lozano‐Cortés, D. , Bode, M. , Almany, G. R. , Harrison, H. B. , Pita, J. , Saenz‐Agudelo, P. , Gereniu, C. , Waldie, P. A. , Peterson, N. , Choat, J. H. , & Berumen, M. L. (2021). Larval dispersal and fishing pressure influence recruitment in a coral reef fishery. Journal of Applied Ecology, 58, 2924–2935. 10.1111/1365-2664.14027

[ece39541-bib-0036] Hamilton, W. D. , & May, R. M. (1977). Dispersal in stable habitats. Nature, 269, 578–581.

[ece39541-bib-0037] Hilbish, T. J. , Sasada, K. , Eyster, L. S. , & Pechenik, J. A. (1999). Relationship between rates of swimming and growth in veliger larvae: Genetic variance and covariance. Journal of Experimental Marine Biology and Ecology, 239(2), 183–193. 10.1016/S0022-0981(99)00009-X

[ece39541-bib-0038] Jablonski, D. (1986). Larval ecology and macroevolution in marine invertebrates. Bulletin of Marine Science, 39(2), 565–587.

[ece39541-bib-0039] Johnson, D. W. , Christie, M. R. , & Moye, J. (2010). Quantifying evolutionary potential of marine fish larvae: Heritability, selection, and evolutionary constraints. Evolution, 64(9), 2614–2628. 10.1111/j.l558-5646.2010.01 20455930

[ece39541-bib-0040] Krueck, N. C. , Ahmadia, G. N. , Green, A. , Jones, G. P. , Possingham, H. P. , Riginos, C. , Treml, E. A. , & Mumby, P. J. (2017). Incorporating larval dispersal into MPA design for both conservation and fisheries. Ecological Applications, 27(3), 925–941. 10.1002/eap.1495 28039952

[ece39541-bib-0041] Kruuk, L. (2004). Estimating genetic parameters in natural populations using the “Animal Model”. Philosophical Transactions: Biological Sciences, 359(1446), 873–890. 15306404 10.1098/rstb.2003.1437 PMC1693385

[ece39541-bib-0042] Lehmann, R. , Lightfoot, D. J. , Schunter, C. , Michell, C. T. , Ohyanagi, H. , Mineta, K. , Foret, S. , Berumen, M. L. , Miller, D. J. , Aranda, M. , Gojobori, T. , Munday, P. L. , & Ravasi, T. (2018). Finding Nemo's Genes: A chromosome‐scale reference assembly of the genome of the orange clownfish *Amphiprion percula* . Molecular Ecology Resources, 19(3), 570–585. 10.1111/1755-0998.12939 30203521 PMC7379943

[ece39541-bib-0043] Leis, J. M. (2010). Ontogeny of behaviour in larvae of marine demersal fishes. Ichthyological Research, 57(4), 325–342. 10.1007/s10228-010-0177-z

[ece39541-bib-0044] Leis, J. M. (2020). Measurement of swimming ability in larval marine fishes: Comparison of critical speed with in situ speed. Marine Ecology Progress Series, 650, 203–215. 10.3354/meps13233

[ece39541-bib-0045] Leis, J. M. , Siebeck, U. , & Dixson, D. L. (2011). How nemo finds home: The Neuroecology of dispersal and of population connectivity in larvae of marine fishes. Integrative and Comparative Biology, 51(5), 826–843. 10.1093/icb/icr004 21562025

[ece39541-bib-0046] Lynch, M. , & Walsh, B. (1998). Genetics and analysis of quantitative traits. Sinauer.

[ece39541-bib-0047] Majoris, J. E. , Catalano, K. A. , Scolaro, D. , Atema, J. , & Buston, P. M. (2019). Ontogeny of larval swimming abilities in three species of coral reef fishes and a hypothesis for their impact on the spatial scale of dispersal. Marine Biology, 166(12), Article 159. 10.1007/s00227-019-3605-2

[ece39541-bib-0048] Majoris, J. E. , Foretich, M. A. , Hu, Y. , Nickles, K. R. , Di Persia, C. L. , Chaput, R. , Schlatter, E. , Webb, J. F. , Paris, C. B. , & Buston, P. M. (2021). An integrative investigation of sensory organ development and orientation behavior throughout the larval phase of a coral reef fish. Scientific Reports, 11(1), 12,377. 10.1038/s41598-021-91640-2 34117298 PMC8196062

[ece39541-bib-0049] Marshall, D. J. , Bonduriansky, R. , & Bussière, L. F. (2008). Offspring size variation within broods as a bet‐hedging strategy in unpredictable environments. Ecology, 89(9), 2506–2517. 10.1890/07-0267.1 18831172

[ece39541-bib-0050] Mengistu, S. B. , Palstra, A. P. , Mulder, H. A. , Benzie, J. A. H. , Trinh, T. Q. , Roozeboom, C. , & Komen, H. (2021). Heritable variation in swimming performance in Nile tilapia (*Oreochromis niloticus*) and negative genetic correlations with growth and harvest weight. Scientific Reports, 11(1), 11,018. 10.1038/s41598-021-90418-w 34040080 PMC8154888

[ece39541-bib-0051] Nanninga, G. B. , & Berumen, M. L. (2014). The role of individual variation in marine larval dispersal. Frontiers in Marine Science, 1, Article 71. 10.3389/fmars.2014.00071

[ece39541-bib-0052] Nanninga, G. B. , & Manica, A. (2018). Larval swimming capacities affect genetic differentiation and range size in demersal marine fishes. Marine Ecology Progress Series, 589, 1–12. 10.3354/meps12515

[ece39541-bib-0053] Parichy, D. M. , Elizondo, M. R. , Mills, M. G. , Gordon, T. N. , & Engeszer, R. E. (2009). Normal table of postembryonic zebrafish development: Staging by externally visible anatomy of the living fish. Developmental Dynamics, 238(12), 2975–3015. 10.1002/dvdy.22113 19891001 PMC3030279

[ece39541-bib-0054] Paris, C. B. , Atema, J. , Irisson, J. O. , Kingsford, M. , Gerlach, G. , & Guigand, C. M. (2013). Reef odor: A wake up call for navigation in reef fish larvae. PLoS One, 8(8), e72808. 10.1371/journal.pone.0072808 24015278 PMC3755995

[ece39541-bib-0055] Pepin, P. (2016). Death from near and far: Alternate perspectives on size‐dependent mortality in larval fish. ICES Journal of Marine Science: Journal Du Conseil, 73(2), 196–203. 10.1093/icesjms/fsv160

[ece39541-bib-0056] Pinsky, M. L. , Palumbi, S. R. , Andréfouët, S. , & Purkis, S. J. (2012). Open and closed seascapes: Where does habitat patchiness create populations with high fractions of self‐recruitment? Ecological Applications, 22(4), 1257–1267. 22827133 10.1890/11-1240.1

[ece39541-bib-0057] Pinsky, M. L. , Saenz‐Agudelo, P. , Salles, O. C. , Almany, G. R. , Bode, M. , Berumen, M. L. , Andréfouët, S. , Thorrold, S. R. , Jones, G. P. , & Planes, S. (2017). Marine dispersal scales are congruent over evolutionary and ecological time. Current Biology, 27(1), 149–154. 10.1016/j.cub.2016.10.053 27989671

[ece39541-bib-0058] Planes, S. , Jones, G. P. , & Thorrold, S. R. (2009). Larval dispersal connects fish populations in a network of marine protected areas. Proceedings of the National Academy of Sciences of the United States of America, 106(14), 5693–5697. 10.1073/pnas.0808007106 19307588 PMC2659712

[ece39541-bib-0071] R Core Team . (2021). R: A language and environment for statistical computing. R Foundation for Statistical Computing. https://www.R‐project.org/

[ece39541-bib-0059] Rideout, R. M. , Trippel, E. A. , & Litvak, M. K. (2004). Paternal effects on haddock early life history traits. Journal of Fish Biology, 64(3), 695–701. 10.1111/j.1095-8649.2004.00335.x

[ece39541-bib-0060] Roberts, C. M. (1997). Connectivity and management of caribbean coral reefs. Science, 278(5342), 1454–1457. 10.1126/science.278.5342.1454 9367956

[ece39541-bib-0061] Roux, N. , Salis, P. , Lambert, A. , Logeux, V. , Soulat, O. , Romans, P. , Frédérich, B. , Lecchini, D. , & Laudet, V. (2019). Staging and normal table of postembryonic development of the clownfish (*Amphiprion ocellaris*). Developmental Dynamics, 248(7), 545–568. 10.1002/dvdy.46 31070818 PMC6771578

[ece39541-bib-0062] Saastamoinen, M. , Bocedi, G. , Cote, J. , Legrand, D. , Guillaume, F. , Wheat, C. W. , Fronhofer, E. A. , Garcia, C. , Henry, R. , Husby, A. , Baguette, M. , Bonte, D. , Coulon, A. , Kokko, H. , Matthysen, E. , Niitepõld, K. , Nonaka, E. , Stevens, V. M. , Travis, J. M. J. , … del Mar Delgado, M. (2018). Genetics of dispersal. Biological Reviews, 93(1), 574–599. 10.1111/brv.12356 28776950 PMC5811798

[ece39541-bib-0063] Salles, O. C. , Saenz‐Agudelo, P. , Almany, G. R. , Berumen, M. L. , Thorrold, S. R. , Jones, G. P. , & Planes, S. (2016). Genetic tools link long‐term demographic and life‐history traits of anemonefish to their anemone hosts. Coral Reefs, 35(4), 1127–1138. 10.1007/s00338-016-1485-1

[ece39541-bib-0064] Shaw, A. K. , D'Aloia, C. C. , & Buston, P. M. (2019). The evolution of marine larval dispersal kernels in spatially structured habitats: Analytical models, individual‐based simulations, and comparisons with empirical estimates. The American Naturalist, 193(3), 424–435. 10.1086/701667 30794444

[ece39541-bib-0065] Simpson, S. D. , Harrison, H. B. , Claereboudt, M. R. , & Planes, S. (2014). Long‐distance dispersal via ocean currents connects Omani clownfish populations throughout entire species range. PLoS One, 9(9), e107610. 10.1371/journal.pone.0107610 25229550 PMC4167857

[ece39541-bib-0066] Watkins, T. B. , & McPeek, M. A. (2006). Growth and predation risk in Green frog tadpoles (*Rana clamitans*): A quantitative genetic analysis. Copeia, 2006(3), 478–488.

[ece39541-bib-0067] West‐Eberhard, M. J. (2003). Developmental plasticity and evolution. Oxford University Press.

[ece39541-bib-0068] Williamson, D. H. , Harrison, H. B. , Almany, G. R. , Berumen, M. L. , Bode, M. , Bonin, M. C. , Choukroun, S. , Doherty, P. J. , Frisch, A. J. , Saenz‐Agudelo, P. , & Jones, G. P. (2016). Large‐scale, multidirectional larval connectivity among coral reef fish populations in the great barrier reef Marine Park. Molecular Ecology, 25(24), 6039–6054. 10.1111/mec.13908 27862567

[ece39541-bib-0069] Wong, M. Y. L. , Uppaluri, C. , Medina, A. , Seymour, J. , & Buston, P. M. (2016). The four elements of within‐group conflict in animal societies: An experimental test using the clown anemonefish. Amphiprion percula. Behavioral Ecology and Sociobiology, 70, 1467–1475. 10.1007/s00265-016-2155-6

[ece39541-bib-0070] Wright, S. (1921). Systems of mating I. The biometric relations between parent and offspring. Genetics, 6, 111–123. 17245958 10.1093/genetics/6.2.111 PMC1200501

