# Supplementary material for: Heritability of dispersal‐related larval traits in the clown anemonefish Amphiprion percula
Source: Ecol Evol. 2022 Nov 27;12(11):e9541. doi: 10.1002/ece3.9541 (PMC9702578; doi:10.1002/ece3.9541)
Supplement: Supplementary file 1 — Appendix S1 [file ECE3-12-e9541-s001.docx]

**S1. Bivariate animal model**

We used a bivariate animal model to analyze larval size and swimming speed simultaneously. The results indicated no genetic covariance between the two phenotypic traits. However, MCMC algorithm convergence was not satisfactory for all variance components (results not shown). Therefore, to facilitate convergence and simplify interpretation of results, we proceeded to analyze the two traits separately.

Model specification

Using the MCMCglmm package in R, the bivariate model was specified in the following way:

cbind(speed,length) ~ trait - 1,

random = ~us(trait):animal + us(trait):clutch,

rcov = ~us(trait):units

Priors

Priors were chosen so that the marginal distribution of the prior for the residual component (R) had a mode of approximately 1, and the marginal distribution of the prior for clutch variance (G_C_) had a mode close to zero. Parameter expansion was used for the prior on additive genetic variance (G_A_) to improve convergence.

G_A_: V = diag(2), nu = 2, alpha.mu = c(0,0), alpha.V = diag(2)*1000

G_C_: V = diag(2)*0.1998, nu = 1.002

R: V = diag(2)*1.998, nu = 1.0002

Covariance estimates

Genetic and residual covariance estimates had credible intervals overlapping zero, but there was evidence for positive clutch covariance. In other words: clutches with larger larvae also tend to have faster larvae (and clutches with smaller larvae also tend to have slower larvae) – but this trend is not driven by relatedness, and the larger individuals within a clutch are not necessarily the faster ones.

Table S1: Posterior modes, credible intervals and effective sample sizes for estimates of covariance between larval size and swimming speed from bivariate animal model. Results in bold had credible intervals that did not overlap zero.

|  | Posterior mode | Posterior  95% credible interval | Effective sample size |
| --- | --- | --- | --- |
| Genetic covariance | -0.0004 | (-0.0497, 0.1678) | 3576 |
| **Clutch covariance** | **0.0103** | **(0.0338, 0.2225)** | 8567 |
| Residual covariance | 0.0107 | (-0.0867, 0.0866) | 5080 |

**S2. Relationship between size and swimming speed**

Consistent with the result above, a linear mixed model of swimming speed as a function of larval size, with clutch ID as a random effect, shows a significant effect of clutch, but no significant effect of larval size.

Table S2: Results of a linear mixed model of larval swimming speed as a function of larval size, with clutch ID as a random effect. The p-values were generated by bootstrapping of likelihood ratios. Significant effect in bold.

|  | Variance | SD | Estimate | Standard Error | t-value | p_bootstrap |
| --- | --- | --- | --- | --- | --- | --- |
| Intercept | NA | NA | -1.21 | 3.08 | -0.39 | NA |
| Larval size | NA | NA | 1.21 | 0.72 | 1.68 | 0.11 |
| **Clutch** | **0.47** | **0.69** | **NA** | **NA** | **NA** | **<0.001** |
| Residual | 4.01 | 2.00 | NA | NA | NA | NA |


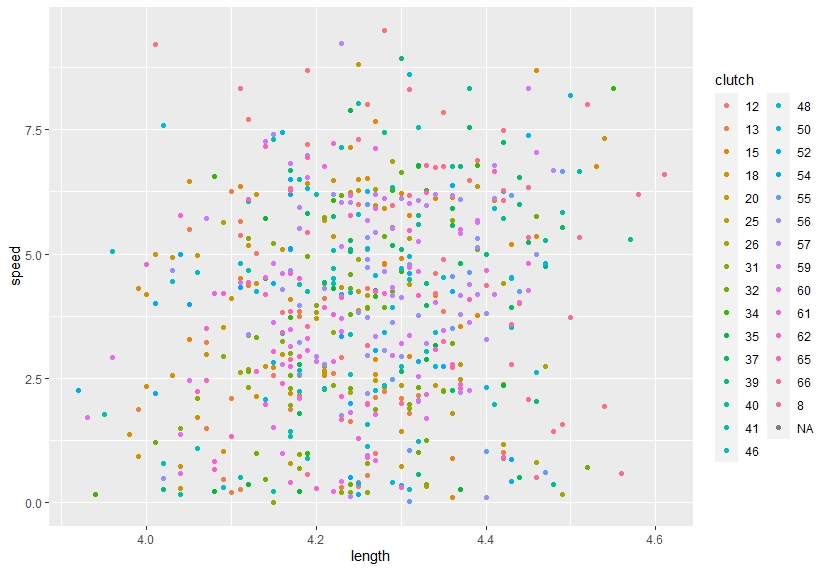


Figure S1: Relationship between length (standard length) and swimming speed (Ucrit). Each point represents one larva; points are colored according to clutch.

**S3. Sensitivity to priors**

We evaluated the sensitivity of our models to the choice of prior by comparing the posterior estimates of additive genetic effect for a range of prior distributions. All priors were univariate inverse-Wishart distributions (equivalent to an inverse-gamma with shape = nu/2 and scale = nu*V/2), with degree of belief parameter nu=0.001 (corresponding to a very flat prior). We chose a range of values for the parameter V, such that priors had modes ranging from 1% to 100% of the total phenotypic variance measured in the trait (total v_P_ was 0.015 for larval size, and 4.6 for swimming speed).

This variation in the prior resulted in a change in the posterior mode of 20% of the total phenotypic variance (for larval size) and 27% of the total phenotypic variance (for swimming speed).


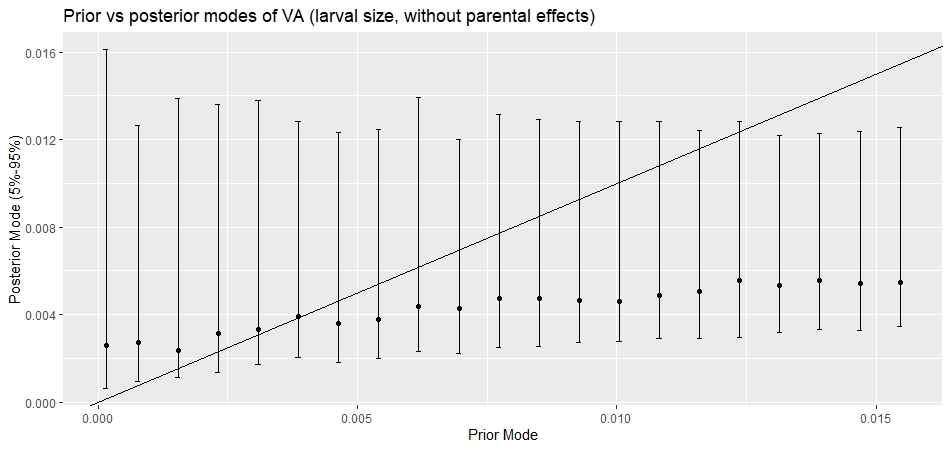


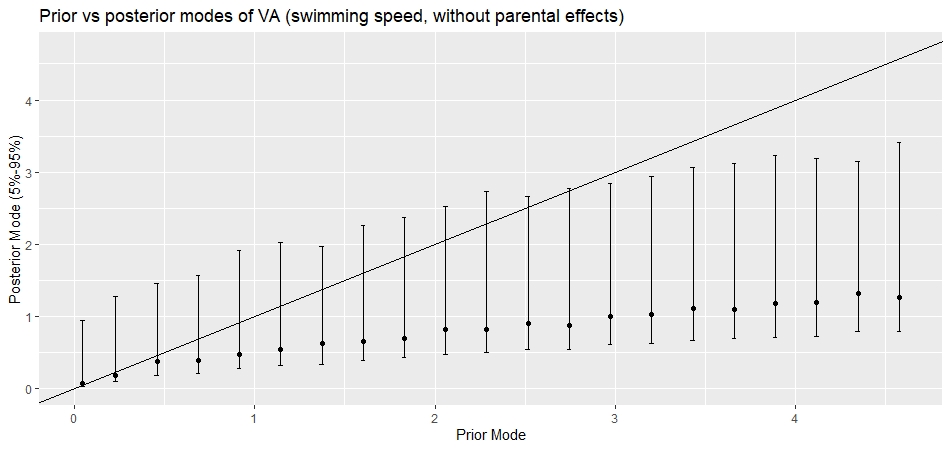


Figure S2: Relationship of prior mode to posterior distribution (mode and 5%-95% intervals) in estimates of additive genetic variance for (A) larval size and (B) swimming speed. Solid diagonal is a 1-1 line.

**Comparison of** **default and alternative priors**

Following are plots of priors and posteriors, and tables of estimates, for all variance components and models. Each row within a figure represents a different choice of prior distributions, as follows:

- **Prior 1**: the default prior reported in the main text. Each variance component has mode equal to 1/k of the total phenotypic variance, where k is the number of variance components. (For example, the total phenotypic variance in Ucrit is 4.58. In the model without parental effects, the priors for each of the three variance components – additive genetic, clutch, and residual – have a mode of 4.58/3 = 1.53.) Inverse gamma distribution with nu = 0.001.
- **Prior 2:** an alternative set of priors with modes close to zero for all variance components. Parameter expanded inverse gamma distribution with V = 1, nu = 1000, α_μ_ = 0, α_V_ = 1.
- **Prior 3:** another formulation of priors with modes close to zero, not using parameter expansion. (The model of swimming speed with this set of priors did not converge, so those results are not included.) Inverse gamma distribution with V = 1, nu = 0.002.

**Standard length, no parental effects**

Additive genetic

Clutch

Residual

Heritability

Prior 1

Prior 2

Prior 3


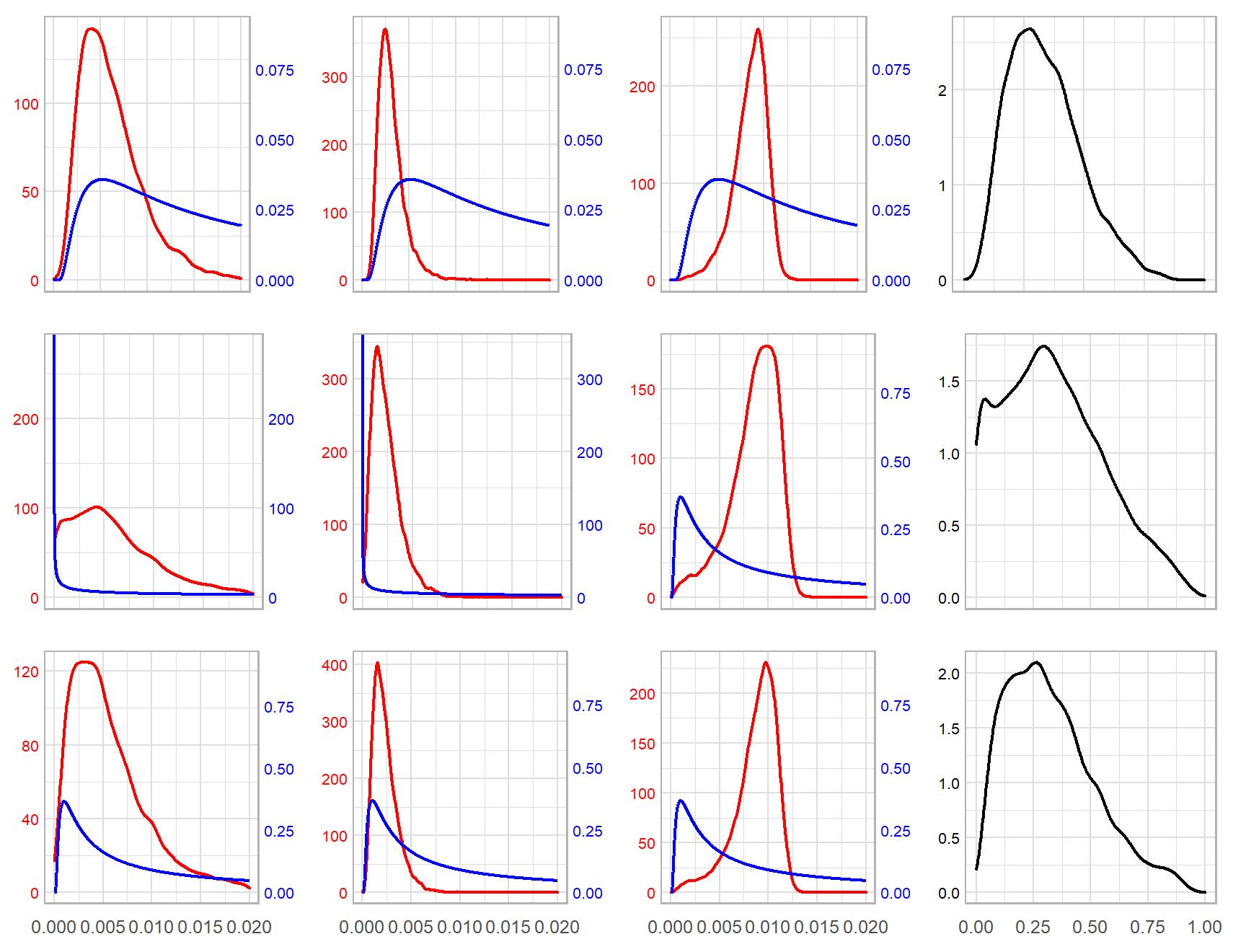


Figure S3: Prior (blue) and posterior (red) estimates of each variance component from a model of larval size with no parental effects. Each column is a different variance component (or heritability), and each row is a different choice of prior (descriptions of priors in text above). All variance components’ x-axes range from 0 to 0.02; the total phenotypic variance is 0.015.

Table S3: Estimates of variance components from a model of larval standard length as predicted by clutch and additive genetic variance. These estimates are generated using the default prior, which distributes total phenotypic variance equally among all components.

| **Component (scaled)** | **Mean** | **2.5%** | **50%** | **97.5%** |
| --- | --- | --- | --- | --- |
| Additive genetic | 0.34 | 0.08 | 0.32 | 0.63 |
| Clutch | 0.17 | 0.06 | 0.16 | 0.30 |
| Residual | 0.49 | 0.22 | 0.51 | 0.72 |

Table S4: Estimates of variance components from a model of larval standard length as predicted by clutch and additive genetic variance. These estimates are generated using the alternative prior, with modes close to zero for all variance components.

| **Component (scaled)** | **Mean** | **2.5%** | **50%** | **97.5%** | **Original Mean** | **Percentage Deviation** |
| --- | --- | --- | --- | --- | --- | --- |
| Additive genetic | 0.33 | 5.98e-9 | 0.31 | 0.74 | 0.34 | -3% |
| Clutch | 0.14 | 1.42e-1 | 0.13 | 0.30 | 0.17 | -18% |
| Residual | 0.53 | 1.61e-1 | 0.55 | 0.81 | 0.49 | +8% |

**Standard length, with parental effects**

Additive genetic

Clutch

Residual

Heritability

Prior 1

Prior 2

Prior 3


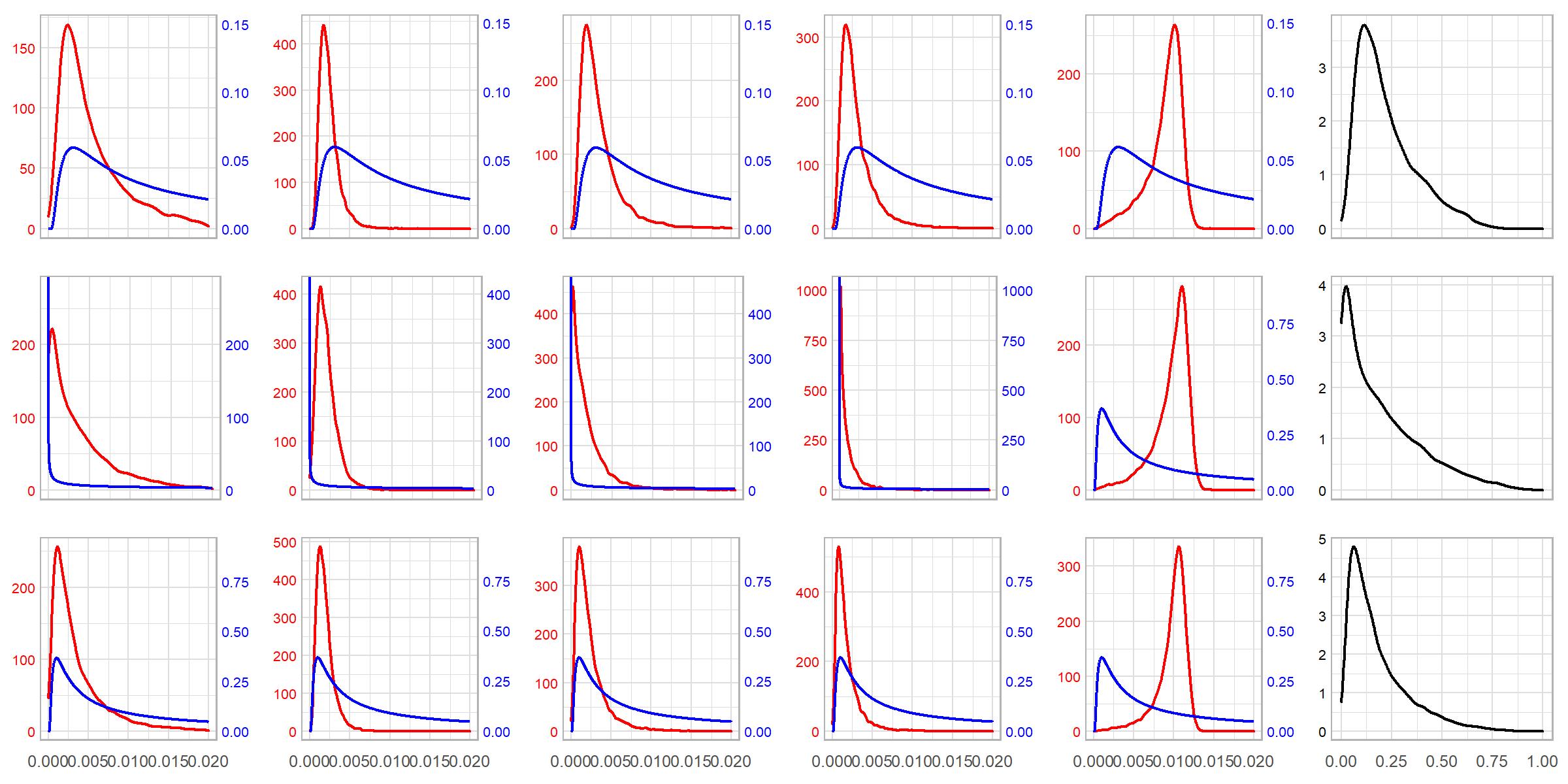


Paternal

Maternal

Figure S4: Prior (blue) and posterior (red) estimates of each variance component from a model of larval size with parental effects. Each column is a different variance component, and each row is a different choice of prior (descriptions of priors in text above). All variance components’ x-axes range from 0 to 0.02; the total phenotypic variance is 0.015.

Table S5: Estimates of variance components from a model of larval standard length as predicted by clutch, additive genetic variance, maternal ID, and paternal ID. These estimates are generated using the default prior, which distributes total phenotypic variance equally among all components.

| **Component (scaled)** | **Mean** | **2.5%** | **50%** | **97.5%** |
| --- | --- | --- | --- | --- |
| Additive genetic | 0.21 | 0.03 | 0.18 | 0.49 |
| Clutch | 0.10 | 0.03 | 0.10 | 0.29 |
| Maternal | 0.15 | 0.03 | 0.13 | 0.31 |
| Paternal | 0.13 | 0.02 | 0.11 | 0.28 |
| Residual | 0.41 | 0.15 | 0.42 | 0.63 |

Table S6: Estimates of variance components from a model of larval standard length as predicted by clutch, additive genetic variance, maternal ID, and paternal ID. These estimates are generated using the alternative prior, with modes close to zero for all variance components.

| **Component (scaled)** | **Mean** | **2.5%** | **50%** | **97.5%** | **Original Mean** | **Percentage Deviation** |
| --- | --- | --- | --- | --- | --- | --- |
| Additive genetic | 0.20 | 3.31e-10 | 0.14 | 0.60 | 0.21 | -4% |
| Clutch | 0.11 | 9.45e-3 | 0.10 | 0.24 | 0.10 | +10% |
| Maternal | 0.09 | 6.06e-11 | 0.07 | 0.28 | 0.15 | -40% |
| Paternal | 0.05 | 2.07e-10 | 0.03 | 0.18 | 0.13 | -60% |
| Residual | 0.55 | 1.96e-1 | 0.58 | 0.80 | 0.41 | +34% |

**Swimming speed, no parental effects**

Additive genetic

Clutch

Residual

Heritability

Prior 1

Prior 2


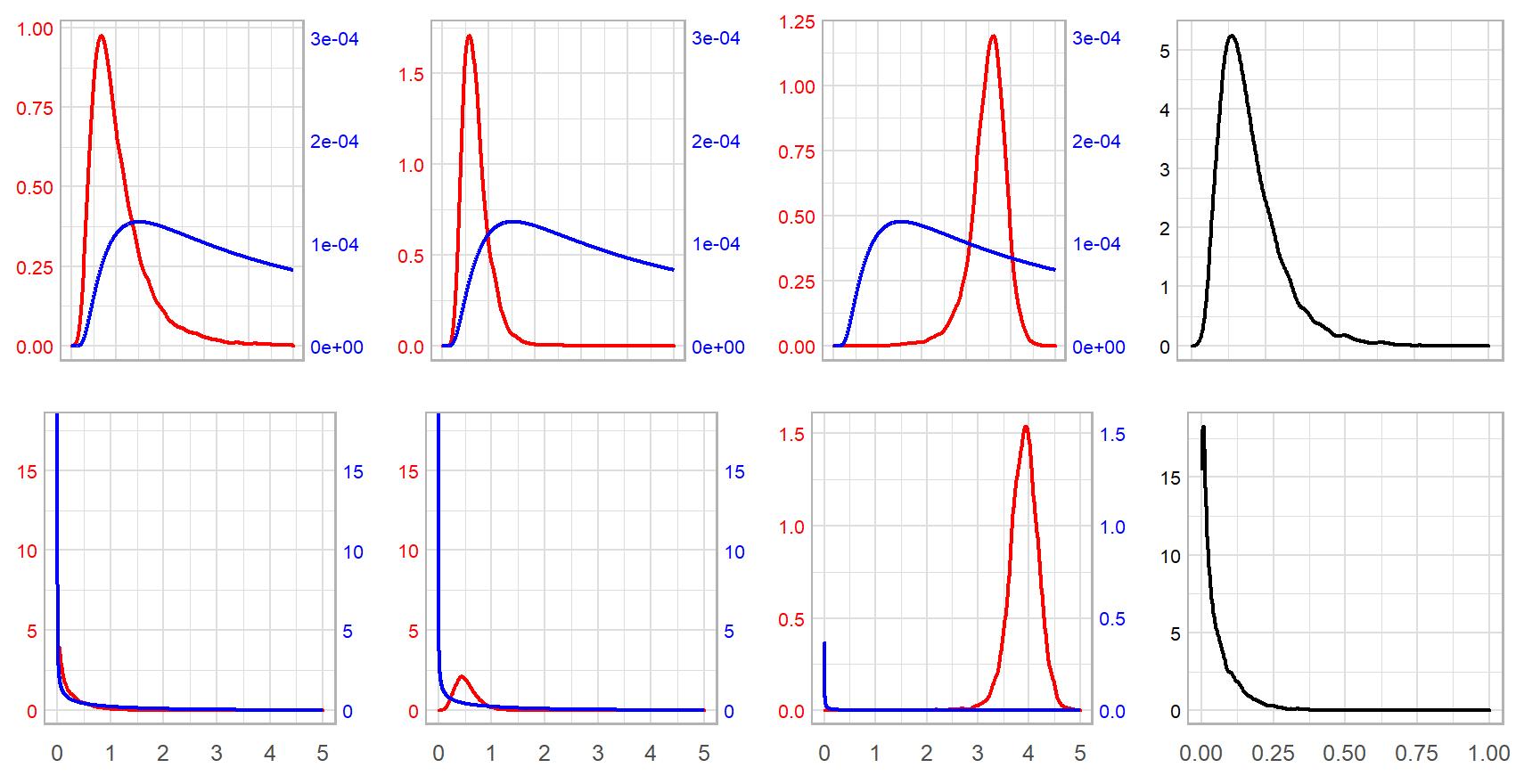


Figure S5: Prior (blue) and posterior (red) estimates of each variance component from the model of swimming speed without parental effects. Each column is a different variance component (or heritability), and each row is a different choice of prior (descriptions of priors in text above). All variance components’ x-axes range from 0 to 5; the total phenotypic variance is 4.58.

Table S7: Estimates of variance components from a model of larval swimming speed as predicted by clutch and additive genetic variance. These estimates are generated using the default prior, which distributes total phenotypic variance equally among all components.

| **Component (scaled)** | **Mean** | **2.5%** | **50%** | **97.5%** |
| --- | --- | --- | --- | --- |
| Additive genetic | 0.19 | 0.05 | 0.17 | 0.39 |
| Clutch | 0.14 | 0.06 | 0.13 | 0.23 |
| Residual | 0.67 | 0.47 | 0.69 | 0.83 |

Table S8: Estimates of variance components from a model of larval swimming speed as predicted by clutch and additive genetic variance. These estimates are generated using the alternative prior, with modes close to zero for all variance components.

| **Component (scaled)** | **Mean** | **2.5%** | **50%** | **97.5%** | **Original Mean** | **Percentage Deviation** |
| --- | --- | --- | --- | --- | --- | --- |
| Additive genetic | 0.05 | 2.25e-10 | 0.03 | 0.18 | 0.19 | -74% |
| Clutch | 0.11 | 3.24e-2 | 0.11 | 0.20 | 0.14 | -21% |
| Residual | 0.84 | 7.05e-1 | 0.85 | 0.94 | 0.67 | +25% |

**Swimming speed, with parental effects**

Additive genetic

Clutch

Residual

Heritability

Prior 1

Prior 2

Paternal

Maternal


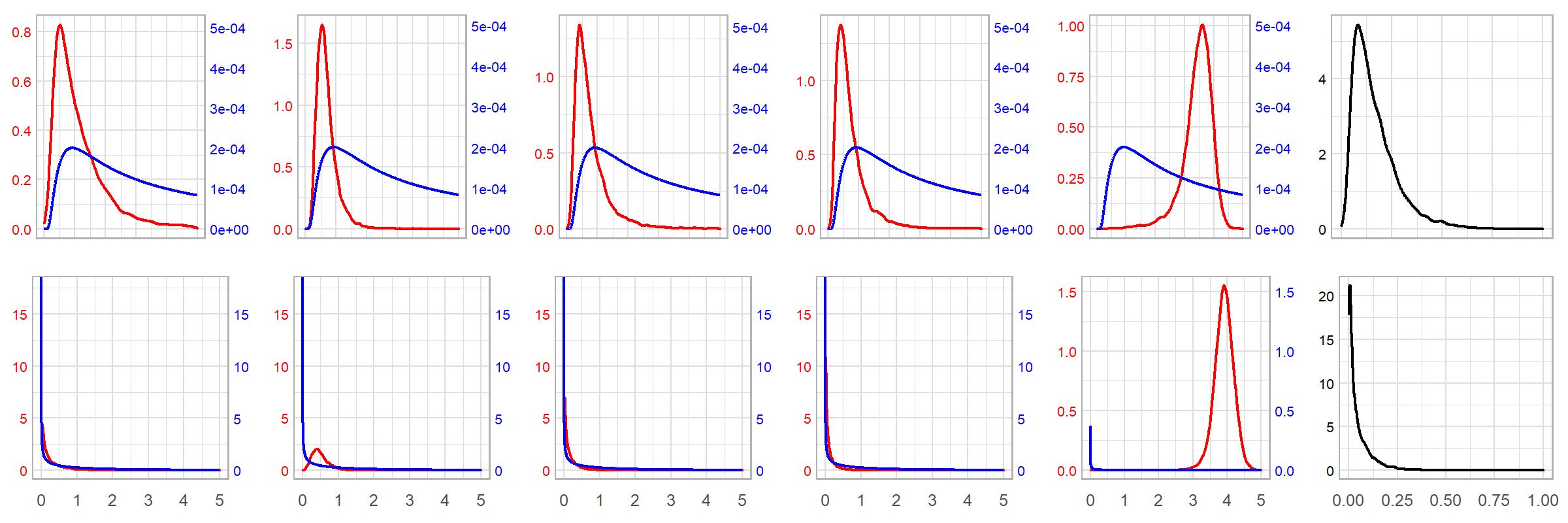


Figure S6: Prior (blue) and posterior (red) estimates of each variance component from the model of swimming speed with parental effects. Each column is a different variance component, and each row is a different choice of prior (descriptions of priors in text above). All variance components’ x-axes range from 0 to 5; the total phenotypic variance is 4.58.

Table S9: Estimates of variance components from a model of larval swimming speed as predicted by clutch, additive genetic variance, maternal ID, and paternal ID. These estimates are generated using the default prior, which distributes total phenotypic variance equally among all components.

| **Component (scaled)** | **Mean** | **2.5%** | **50%** | **97.5%** |
| --- | --- | --- | --- | --- |
| Additive genetic | 0.16 | 0.03 | 0.14 | 0.38 |
| Clutch | 0.10 | 0.03 | 0.09 | 0.18 |
| Maternal | 0.11 | 0.02 | 0.09 | 0.24 |
| Paternal | 0.10 | 0.02 | 0.09 | 0.22 |
| Residual | 0.52 | 0.29 | 0.54 | 0.73 |

Table S10: Estimates of variance components from a model of larval swimming speed as predicted by clutch, additive genetic variance, maternal ID, and paternal ID. These estimates are generated using the alternative prior, with modes close to zero for all variance components.

| **Component (scaled)** | **Mean** | **2.5%** | **50%** | **97.5%** | **Original Mean** | **Percentage Deviation** |
| --- | --- | --- | --- | --- | --- | --- |
| Additive genetic | 0.05 | 9.98e-11 | 0.02 | 0.17 | 0.16 | -69% |
| Clutch | 0.10 | 2.32e-2 | 0.09 | 0.18 | 0.10 | 0% |
| Maternal | 0.03 | 1.06e-12 | 0.01 | 0.10 | 0.11 | -73% |
| Paternal | 0.02 | 1.07e-10 | 0.01 | 0.07 | 0.10 | -80% |
| Residual | 0.81 | 6.60e-1 | 0.82 | 0.93 | 0.52 | +56% |
